# Ethical challenges among registered dietitians in oral nutritional supplement prescription

**DOI:** 10.1177/09697330251350390

**Published:** 2025-06-25

**Authors:** Susanna Pohjola, Åsa Grauman, Anna T Höglund, Elin Lövestam, Margaretha Nydahl, Evelina Liljeberg

**Affiliations:** Uppsala University

**Keywords:** Ethical challenges, ethical principles, oral nutritional supplements, qualitative research, registered dietitian

## Abstract

**Background:**

Ethical considerations are an inherent part of all clinical decision-making, yet the specific ethical challenges faced by registered dietitians [RDs] remain underexplored.

**Aim:**

Explore RDs ethical challenges in oral nutritional supplement prescription [ONS].

**Research Design:**

A qualitative descriptive research design.

**Participants and Research Context:**

Thirteen RDs working in Swedish primary care (*n* = 7) and hospital settings (*n* = 6) were recruited via purposive sampling through the professional association. Data were collected through individual semi-structured interviews (Oct 2019–Apr 2020) and analyzed using systematic text condensation [STC], containing four iterative steps.

**Ethical Considerations:**

The Swedish Ethical Review Authority [Reference No. 2019-01198 and 2023-06903-02] approved the study protocol and all participants provided written informed consent.

**Findings:**

Through STC, two main categories were identified: (1) Structural prerequisites for equitable and accessible care and (2) Navigating professional judgment and the patient’s wishes. Registered Dietitians described ethical challenges related to justice, such as patients in rural areas lacking transport to care facilities or ONS home delivery. Autonomy-related challenges involved persuading vulnerable patients who were dependent on others for nutrition support. Registered dietitians also faced ethical challenges related to beneficence and non-maleficence, when balancing ONS prescription with concerns about replacing regular meals.

**Conclusions:**

Registered dietitians described ethical challenges, such as discomfort due to financial or systemic barriers, and using paternalistic persuasion. These challenges may lead to moral distress, highlighting the need for ethics support to reduce distress and promote well-being. Healthcare systems should also ensure equitable access and clarify ONS prescription guidelines.

## Introduction

An ethical dimension is inevitably inherent in all decision-making in healthcare. However, this aspect remains underexplored within the relatively young dietetic profession, which has taken on an increasingly central role in healthcare.

Ethical decision-making is a core healthcare competency, guided by professional codes, grounded in bioethical principles of autonomy, beneficence, non-maleficence, and justice.^1–8^ These principles offer a framework for navigating complex situations, such as balancing patient autonomy with promoting well-being.^9^ However, applying them in daily practice is not always straightforward. There is a gap between ethical theory and clinical reality, particularly in areas like end-of-life nutrition support. This highlights the importance of emphasizing ethical principles in everyday clinical practice and the need for empirical research on how registered dietitians [RDs] navigate ethical challenges.^10^

Ethics offers a structured lens for addressing such dilemmas, distinguishing itself from morality, which is more individually and culturally shaped.^8,9,11,12^ Yet these terms are often used interchangeably, leading to conceptual inconsistencies.^11–13^ In this study, ethical challenges are defined as situations where professionals must choose between two morally valid options or are hindered from acting ethically due to institutional or contextual constraints.^14^

Malnutrition increases morbidity, mortality, and reduces quality of life.^15^ Registered Dietitians play a central role in its prevention and treatment through interventions^16^ such as texture-modification, fortification, frequent energy and nutrient dense meals, and oral nutritional supplements [ONS],^17^ which support individuals unable to meet nutritional needs through food alone.^18,19^ In Sweden, RDs are primarily responsible for ONS prescription,^20^ though this varies internationally.^21,22^

While ethical issues in nutrition therapy have been explored in contexts like artificial nutrition at the end of life and food choice autonomy in long-term care,^23–27^ limited empirical attention has been given to RDs’ experiences of ethical challenges in ONS prescription for patients who eat orally but remain at nutritional risk. These patients present complex clinical and ethical considerations that often fall outside of the more visible, high-stakes contexts typically studied.

**Figure 1.**
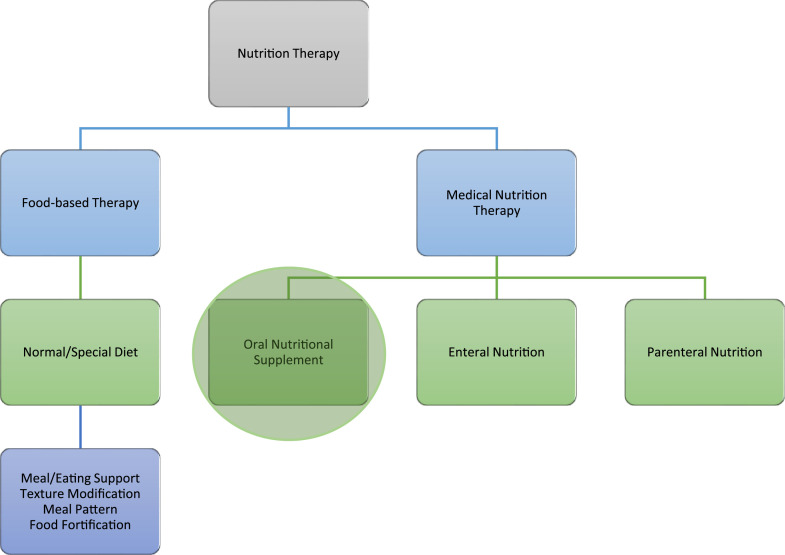
Concept map illustrating areas within nutrition therapy.^30^

In practice, RDs must tailor decisions not only to clinical guidelines, which promote “food first” approaches before ONS (Figure 1),^28–30^ but also to individual patient needs, palliative goals, public health priorities, and organizational constraints.^16,31–34^ Such decisions are often made under time constraints, with limited staffing and interdisciplinary coordination.^35,36^ These factors, compounded by broader commercial and sociopolitical influences, contribute to the ethical complexity of dietetic practice.^37^

Despite the centrality of RDs in nutritional care, little is known about how they navigate ethical challenges in clinical practice, particularly in relation to ONS prescription. To address this gap, the aim of this study is to explore RDs’ ethical challenges in ONS prescription.

## Methods

### Study design

This qualitative descriptive study is a secondary analysis of a previous dataset. It focuses on RDs’ ethical challenges in ONS prescription and applies Systematic Text Condensation [STC] for analysis, as described by Malterud.^38^

### Participants and research context

The study is based on individual semi-structured interviews with 13 Swedish RDs (mean age 36 ± 12 years; mean experience 10 ± 10 years), six working in hospitals and seven in primary care. All routinely prescribed ONS to patients with various conditions associated with malnutrition, such as neurological diseases, cancer and gastrointestinal disorders. Participants were purposively recruited via the Swedish Association of Registered Dietitians’ newsletter and Facebook group, enabling access to diverse clinical contexts and broad professional experience. Interviews were conducted by the last author, at the time a PhD student, between October 2019 and April 2020 under experienced qualitative research supervision. Each interview lasted 39–68 minutes (mean 55) and was recorded and transcribed verbatim. The interview guide is available in the appendix of the primary study.^39^ Participants were informed about the interviewer’s background and the study purpose. The interviewer had prior acquaintance with two participants as former colleagues, but no supervisory or hierarchical relationship existed.

Data collection concluded when information power was deemed sufficient.^40^ Since this is a secondary analysis, readers are referred to a previous publication for further details on recruitment, demographics and data collection procedures.^39^

In Sweden, healthcare is publicly funded and ONS prescribing and product selection are managed regionally. Subsidized ONS are available for patients with or at risk of malnutrition, though subsidy levels vary by region.^41^ ONS is also available without prescription, but patients with subsidies typically receive a 1-month supply delivered to their home or a collection point.

### Data analysis

During the initial analysis, themes related to ethical challenges, decision-making, and moral distress were identified, though these were not the primary focus at that time.^39^ These themes were flagged for further investigation. In this secondary analysis, the research team was expanded to include ethics expertise, allowing for a more in-depth exploration of these ethical challenges.

STC is a pragmatic, stepwise method for thematic cross-case analysis of qualitative data, suited for systematically exploring participants’ experiences and perspectives.^38^ The software Taguette aided data organization.^42^ The analysis was conducted by an interdisciplinary team, comprising six researchers with expertise in nutrition, dietetics, and ethics. Among them, three are RDs with clinical experience, one doctoral student is a registered nurse specialized in primary healthcare, and two researchers have specific expertise in ethics. This diverse expertise enriched the analysis, ensuring a comprehensive and multifaceted approach.

The STC analysis involved four steps.^38^ Initially, all investigators reviewed portions of the transcripts to identify preliminary themes related to the study’s focus on ethical challenges in ONS prescription. Relevant meaning units were identified, coded, and grouped into code groups, decontextualizing the data. Next, nuances within each group were identified as subgroups. We revisited the initial meaning units in each subgroup, writing condensates that retained the original terminology used by participants. These condensates formed the basis for the analytical text, which synthesized and reconceptualized the data into coherent, multi-vocal narratives grounded in the original context. Categories were developed to offer brief and expressive statements of the most significant interpretations. Reflexivity was emphasized to ensure interpretations remained grounded in the original data, allowing for an iterative analysis process that revisited and validated findings against the transcripts to ensure thoroughness.^38^ The phenomenological approach aimed to set aside preconceptions^38,43^ and focus on the RDs’ perspectives.

The first author primarily conducted the data analysis, with regular meetings held with the last author to refine themes, code groups, subgroups, condensates, and the analytical texts. All authors contributed to the manuscript’s development and to a comprehensive and rigorous analysis,^44^ providing regular feedback and review, with the last author offering continuous guidance throughout the writing process. Preliminary themes, code groups, subgroups, and categories are detailed in Table 1. To deepen the understanding, results were interpreted through an ethical lens, drawing on the bioethical principles of autonomy, beneficence, non-maleficence, and justice,^8^ as addressed in the Discussion section.

**Table 1.** Overview of the analysis.

Step 1: Preliminary themes	Step 2: Code groups	Step 3: Subgroups	Step 4: Categories
Patient participation Structural prerequisites Interprofessional collaboration and responsibilities	(1) From production to delivery of ONS	(1A) Meeting patients’ taste preferences	1. Structural prerequisites for equitable and accessible care
		(1B) Lack of guidelines gives space to subjective assessments	
		(1C) The delivery system and the environment	
	(2) Not everyone gets access to ONS	(2A) Varying possibilities for physical interactions	
		(2B) the cost for the patient	
	(3) Communication between governing bodies and other healthcare professionals	(3A) Need for cross-border collaboration to care for the patient as a whole	
		(3B) Over stepping professional boundaries	
		(3C) Prescriptions due to lack of social resources	
	(4) The dance with the patient	(4A) Inform to comply or support to adhere?	2. Navigating professional judgment and the patient’s wishes
		(4B) Letting patients decide	
	(5) Limited ability to partake in the care	(5A) Steering interventions	
		(5B) Vulnerable patients need to rely on others support	
	(6) The RD’s conclusion and the patient’s resistance	(6A) Safety and simplicity	
		(6B) Prescription consequences	

### Ethical considerations

The Swedish Ethical Review Authority [Reference No. 2019-01198 and 2023-06903-02] approved the study protocol and all participants provided written informed consent. The authors have adhered to the guidelines for responsible research publication and the ethical standards recommended by the Committee on Publication Ethics.^45^ The documentation and reporting of this research adhered to the Consolidated Criteria for Reporting Qualitative Research.^46^

## Results

Several areas where RDs encounter ethical challenges in their daily work were identified in the analysis. The results were organized into two main categories with three sub-categories, respectively (see Table 2).

**Table 2.** Results.

Categories	Sub-categories		
1. Structural prerequisites for equitable and accessible care	Logistics of ONS	Optimal ONS treatment does not reach everybody	Allocation of responsibilities
2. Navigating professional judgment and the patient’s wishes	Balancing support and patient responsibility	Persuasion and relying on others	Drawbacks of ONS prescription

### Category 1: Structural prerequisites for equitable and accessible care

Ethical challenges in this category highlight how the interests of companies and governing bodies can conflict with ensuring fairness for patients. The RD’s ability to provide equitable and accessible care of high quality were affected by structural prerequisites within the healthcare system, but also aspects related to the patients’ living conditions.

#### Logistics of ONS

Registered Dietitians described several ethical challenges during the prescription of ONS related to the interpretation of nutrition intervention guidelines and the patient receiving the product. They sometimes struggled with determining if a patient fulfilled the criteria for ONS prescription. Some reported a lack of clarity in the guidelines, and that local or national guidelines allowed for some degree of interpretation, leading to subjective assessments. Another example of external factors influencing the availability of ONS is that ONS companies focus on producing economically profitable flavors. Some RDs highlighted this as an issue, particularly when it conflicted with patients’ preferences for Nordic flavors, since taste was described as a crucial factor for patient acceptance.

“We have been asking for lingonberry, blueberry and lemon flavors, both in juice-based and milk-based ONS […], but Sweden is […] such a small market, and these are big companies […] we used to have lingonberry, blueberry and rose hip flavors […] very popular, high in calories, but […] we ask the representatives every time they come […] but I know, we know […] Sweden is too small” Registered dietitian B, hospital.

Challenges also arose related to the ONS delivery system, which varied by region. Most regions offered home delivery, while others required patients to fetch products from a collection point. Some RDs noted patients’ reluctance to store 1 month’s supply at home. Changed patient preferences also led to wastage, which concerned both RDs and patients, due to potential negative environmental impacts.

#### Optimal ONS treatment does not reach everybody

Some RDs implied that structural prerequisites sometimes prevented ONS from reaching the patients who needed them. They described ethical challenges in striving to provide ideal nutritional care, while navigating organizational and financial constraints. For example, some patients who were considered to benefit from ONS could not afford it. This weighed on the conscience of some RDs, as one RD illustrated:

“Then it’s like with all systems that sometimes someone suffers and it […] is really difficult to manage […] if I could wish for something […] it would be to be able to remove the cost for those who really can’t afford it […] it would be kind of nice to be able to […] offer them equitable care like that. So that they could still get ONS” Registered dietitian F, hospital.

The RDs described that the optimal way to provide high-quality nutritional care was through physical meetings to discuss ONS therapy. However, the ability to offer this ideal situation varied based on the healthcare system’s structure and the patient’s ability to visit the healthcare center.

“You don’t always meet them […] except for some COPD patients […] who can manage it - those you meet, but then there are the elderly and the very sick […] who aren’t always able to come […] it’s difficult for them actually, if they can’t drive […] There’s a bus stop here but […]only three buses per day or so, […] so […] in which case they have to be able to walk, I don’t know, maybe five hundred meters” Registered dietitian L, primary care

In contrast, some RDs in primary healthcare described the possibility of making home visits, allowing them to offer more appropriate nutritional care to vulnerable patients unable to travel to the healthcare center. Another RD in primary care described:

“If they are very unwell, then you have to make a home visit […] and these can be people who are wheelchair users, those confined to bed, or so confused by Alzheimer’s that visiting the healthcare center is too overwhelming […] and then it’s nice for them to have this visit at home […] in peace and quiet” Registered dietitian G, primary care.

#### Allocation of responsibilities

Registered dietitians described challenges in dividing responsibilities with other healthcare professionals and governing bodies. For instance, they described how the norm of using regular food as the first strategy before introducing ONS could conflict with the patient’s expectations, sometimes created by other healthcare professionals. Several RDs described how patients were promised a prescription by their physician, before their appointment with the RD. The RDs also stated that they have cutting-edge expertise in the subject and that treatment with ONS is “*not just about unscrewing the cap and serving the ONS*,” Registered dietitian G, primary care. It is important to meet the patients, give them individualized treatment, and provide follow-ups to achieve good compliance.

Registered dietitians reported varying levels of collaboration with governing bodies, such as municipal home care services. They emphasized that effective collaboration, including educating and supporting home care staff, fostered open communication and mutual confidence, ensuring alignment on what needed to be done and how, which ultimately created a seamless care experience. However, challenges included frequent staff turnover, less committed staff, and communication difficulties due to numerous home care companies operating in the municipality. Registered dietitians also faced challenges when patients lacked healthcare unit affiliation, causing them to fall through the cracks with no governing body taking responsibility for their care. In these situations, RDs had to decide whether to prescribe ONS despite the clinic’s guidelines, which only allowed for treatment of affiliated patients. The situation became even more complex in cases where no physician was available to guide the decision, leaving the RD to make the call independently. As one RD described:

“As long as they have some follow-up […] in X, then […] that RD should take them. However, she doesn’t treat palliative patients, only those receiving curative treatments. But then the palliative patients end up with us, even though we don’t have a physician here, only […] medical testing [nurse-based service] […] and then you have to rely on what your conscience says that day. Should I be tough or should I be kind?” Registered dietitian B, hospital.

The quote highlights the RD’s internal struggle between adhering to professional guidelines and helping the patient. The ethical tensions arise from the lack of clear directives regarding patient responsibility, leaving the RD to rely on personal conscience when deciding whether to prescribe ONS, even though doing so exceeds the formal role of her healthcare facility.

The RDs reported that the pressing time constraints on other healthcare professionals made ONS an easier alternative to food-based strategies. However, the opinions of the RDs varied regarding the prescription of ONS in response to system shortcomings. While some felt compelled to use ONS as a band-aid solution due to limited resources, others argued that ONS should not be the solution when patients simply needed help with tasks such as serving meals or cooking, especially if they had no difficulties eating regular food.

### Category 2: Navigating professional judgment and the patient’s wishes

Ethical challenges in this category relate to RDs endeavors to respect patients’ wishes, which encompass both practical aspects such as preferences and choices, and a deeper desire for self-determination, while ensuring effective ONS therapy. In cases of limited autonomy, more directive interventions were described, highlighting the tension between patient wishes and professional judgment in clinical decision-making.

#### Balancing support and patient responsibility

Some RDs described supporting patients to improve their nutritional status, while promoting independence. The RDs advocated for patient involvement and supported their autonomy, although they sometimes faced challenges when respecting the patient’s self-determination and integrity conflicted with promoting the patient’s best interests from a nutritional perspective. For example, some RDs confronted patients who reported a higher ONS intake than they actually had. This could, according to RDs, lead to patients feeling offended and disbelieved, creating a difficult situation for the RDs. Patient autonomy was sometimes prioritized over strictly adhering to nutritional guidelines, despite knowing it may not lead to the best nutritional outcomes:

“Many older ladies with COPD have had a lifestyle where their aim was to lose weight […] and now when they’ve finally become thin […], which is their ideal, the mean RD comes along and wants them to take ONS so they get fat […] so, this may need a motivational intervention […] to help them accept […] the idea that this weight loss is involuntary and not healthy. Occasionally, they have agreed to take ONS and then returned them all […] They don’t want to get fat and that’s their choice […] You can’t take things personally […] Because I’ve noticed that often the ideal of older ladies is to be […] thin, they don’t want to gain weight even if they need to […] or at least keep their weight stable” Registered dietitian G, primary care.

This quote highlights the tension between respecting patients’ wishes, such as their desire to maintain a certain weight, and the RD’s responsibility to guide them toward optimal nutritional status.

#### Persuasion and limited autonomy

Some RDs described challenges when patients could not participate in their care, making them reliant on others for proper ONS treatment. When relatives or staff neglected to administer ONS, non-compliance occurred through no fault of the patient. Although RDs aimed to involve patients, autonomy was limited among children, those with cognitive impairments, and patients in acute settings, complicating efforts to ensure their wishes. In these cases, more directive interventions were sometimes necessary, requiring RDs to override patient autonomy to ensure proper ONS treatment:

“You have to trick people with dementia a bit (whispers) […] It’s easier if they think it’s a medicine. Then it can be easier to motivate them to drink it […] some elderly with dementia are grumpy, they are so stubborn, you have to […] come up with something […] and so I usually say that […] Sometimes I talk to a relative or the home care staff and tell them to tell the patient ‘it’s medicine, the doctor has said you have to drink this’. It’s a bit wicked, but then […] it’s just to motivate them to drink it.” Registered dietitian L, primary care.

This quote highlights the conflicting values between improving nutritional status and preserving the patient’s dignity through honesty, acknowledging that these values may not always be achievable simultaneously.

#### Drawbacks of ONS prescription

Some RDs highlighted potential drawbacks of prescribing ONS. While RDs prescribed ONS with the intention of acting in the best interest of the patient and honoring their wishes, some RDs regarded prescribing ONS as taking away some of the patient’s independence. Another consequence was the strong reactions from patients when RDs decided that ONS were no longer a necessary part of the nutrition intervention, leading to some dissatisfied patients. Sometimes ONS became a substitute for regular food, leading to the patient missing the social aspects of eating. The challenge of navigating situations when the meaning of ONS had transitioned to something more than energy and nutrients for patients was described. Consequently, weaning patients off the ONS was described as challenging:

“If they’ve had them for a long time and it’s kind of a part of their everyday life […] Then yes, it becomes more difficult to discontinue them […] absolutely. I Think it’s this sense of security […]it becomes part of their everyday life, I think […] It’s as if someone says ‘no, you can’t drink coffee anymore’ or ‘you have to now pay double the amount” Registered dietitian I, primary care.

This quote illustrate how discontinuing ONS can conflict with the patient’s wishes, even when the RD deems it medically unnecessary due to improved nutritional status.

## Discussion

Throughout the analysis, various challenges related to RDs’ daily practice and the prescription of ONS were identified. In the following section, these challenges are explicitly linked to the bioethical principles of autonomy, beneficence, non-maleficence, and justice, as outlined by Beauchamp and Childress,^8^ and the potential consequences will be discussed to further illuminate the ethical implications of RDs’ work.

### Competing bioethical principles in ONS prescription

#### Autonomy and beneficence

Ethical challenges arise when principles like autonomy and beneficence conflict, as professionals navigate between clinical decisions and patient preferences or cultural norms.^7,9,47^ This was evident in the present study, where RDs sought to improve nutritional status through ONS prescriptions, while balancing the aim of beneficence and avoiding harm with respecting patients’ right to self-determination. For example, an elderly woman resisted ONS, as gaining weight contradicted her lifelong pursuit of thinness. In such cases, RDs faced the challenge of respecting autonomy or intervening to prevent harm, a balance that could strain patient-provider relationships, highlighting the delicate balance between promoting health and respecting individual autonomy.^48^

Shared decision-making (SDM) should guide healthcare professionals to ensure that care decisions align with patient preferences.^48^ However, when SDM is not possible, paternalism may be justified in the patient’s best interest,^9,48^ provided it respects the patient’s values and supports a trusting care relationship.^48^

In cases where autonomy could not be exercised, such as with dementia, RDs described how paternalistic approaches were sometimes necessary to improve nutritional status. Still, interventions should consider the patient’s overall well-being, ideally defined by the patient, and strive for collaboration whenever possible. This tension reflects ongoing ethical debates around autonomy and paternalism in healthcare.^49,50^

One RD explained convincing patients with dementia that ONS were “medicine,” raising concerns about deception, reflecting real-world challenges where beneficence is prioritized over autonomy.^48^ Such interventions are intended to promote well-being, not misleadning,^51^ underscoring ethical tensions in decision-making regarding ONS.

#### Justice and non-maleficence

Previous research highlights how organizational interests sometimes overrule patient preferenses.^16^ Additionally, ONS usage has been shown to reduce healthcare costs, further complicating ethical considerations related to justice and non-maleficence.^52^ In our study, ONS were sometimes used as a band-aid solution to a system failure, such as time constraints faced by other healthcare professionals hindering food-first strategies. While this approach reduced immediate harm, it potentially contributed to long-term issues, such as social isolation and inequitable access to sustainable, food-based interventions. Substituting ONS for regular meals could undermine the social and psychological benefits of regular meals, a critical factor for older adults at risk of malnutrition.^53^

Prescribing ONS without follow-up can lead to unintended harm.^33^ Many older adults experience health inequities related to nutrition and due to the social determinants of health, such as economic instability and access to quality health care.^53^ In situations when patients could not afford the treatment, RDs in our study felt tempted to bend the rules even though prescribing ONS at no cost was against regulations. Treating patients with limited access to healthcare facilities due to their geographical location was also described as challenging. These situations forced RDs to adapt interventions based on patients’ economic and geographical circumstances, compromising beneficial care and raising ethical concerns about fairness. Moreover, subjective judgments of ONS necessity introduce inequities, since RDs’ interpretations of patient circumstances as well as medical condition vary, despite guidelines aiming for consistency.^28–30^

#### Moral distress and ethical competence

When healthcare professionals are unable to act in accordance with their ethical convictions due to institutional constraints, competing values or systemic barriers,^35,36^ they may experience moral distress.^47,54^ This well-documented phenomenon arises from the psychological and emotional impact of being constrained from doing what one believe is ethically right,^47,54–56^ often resulting in feelings of frustration, anger, anxiety, guilt and shame. Over time, moral distress can contribute to burnout and decreased job satisfaction.^56,57^ Moral distress tends to lead to compensatory behaviors, such as going above and beyond what the professional role requires. During the covid-19 pandemic, healthcare professionals compensated for organizational shortcomings by working with limited resources, working overtime, and assuming unclear or new roles. Such compensatory actions often stem from the moral distress of feeling powerless to act in accordance with their moral and professional standards.^58^

Although RDs in this study did not explicitly discuss the emotional toll to the same extent, their discomfort at not being able to reduce costs for patients in need or feeling ashamed about certain interventions used with vulnerable patients, such as those with dementia, suggests potential signs of moral distress. Moral distress could possibly arise therefore when being torn between following regulations and helping patients who could not afford ONS. Additionally, using ONS as a temporary fix for system failures in the healthcare system could contribute to moral distress, where the RDs felt they sometimes needed to do more than their professional role required.

Competence and professional development in practice are key elements in preventing harm.^4^ Lifelong learning in ethics, through continuing education in ethical reflection and decision-making, may help RDs build and strengthen their ethical competence, equipping them to navigate ethical challenges in daily practice,^2,47^ such as those presented in this study, and potentially mitigate moral distress.^47^

### Strengths and limitations

This study on the ethical challenges in ONS prescription among RDs has both limitations and strengths that impact its validity and transferability.^44^ There is consistency between the data presented and the findings. The systematic text condensation of the interview data revealed patterns and themes that aligned with the study’s aim to explore RDs’ experiences of ethical challenges when prescribing ONS. The findings were supported by quotes from participants, which strengthened the account of their experiences. A key limitation is that it is based on a secondary analysis. The original interview questions were designed to explore general dietetic practices, not ethical challenges specifically, which may have limited the data’s depth in addressing such issues, affecting the internal validity.^44^ However, secondary analysis offers strengths, such as reducing response bias, as participants were not directly asked about sensitive topics. This approach can also reveal new themes not considered in the original research, while also being resource efficient. A major strength is the involvement of a multidisciplinary research team. The first author, who primarily conducted the data analysis, does not have a background in dietetics, which may reduce the potential for bias stemming from personal or professional experiences within the field. The team, with expertise in nutrition, dietetics, and ethics, enriched the analysis by bringing varied perspectives. This diversity enhanced the study’s credibility^44^ and depth, ensuring a comprehensive understanding of ethical challenges in ONS prescription. Potential drawbacks of a RD interviewing peers and how this influences the responses, are discussed in the first article.^39^

## Conclusions

This study highlights challenges in RDs’ daily work when prescribing ONS, which when viewed through the lens of bioethical principles, can be linked to ethical challenges and moral distress. These challenges arise not only from the RD-patient relationship but also from broader systemic issues within health care. Our findings show that RDs often struggle to balance patient autonomy with the need to ensure effective and beneficial treatment. Additionally, RDs experienced challenges in navigating the provision of equitable nutrition care and reducing harm by prescribing ONS for patients who had been missed by the healthcare system. Such ethical challenges may increase the risk of moral distress among RDs, underscoring the need for structured ethics support in clinical practice. Further research is suggested to explore how such support can be developed and integrated into dietetic practice to strengthen ethical competence and promote sustainable professional well-being. To address systemic issues, healthcare systems should ensure equitable access to nutrition care and clarify ONS prescription guidelines.

## Data Availability

Due to ethical and legal considerations, the dataset generated and analyzed during the current study is not publicly available. The data contains sensitive information that could compromise participant privacy and confidentiality.*
